# 3-(2-Pyrid­yl)-5-(4-pyrid­yl)-4-(*p*-tol­yl)-1*H*-1,2,4-triazole

**DOI:** 10.1107/S1600536809016432

**Published:** 2009-05-07

**Authors:** Lu-Tong Yuan, Hai Zhang, Zuo-Xiang Wang, Zhi-Rong Qu

**Affiliations:** a Benxi Health School of Liaoning, Liaoning 117022, People’s Republic of China; b Ordered Matter Science Research Center, College of Chemistry and Chemical Engineering, Southeast University, Nanjing 210096, People’s Republic of China

## Abstract

In the mol­ecule of the title compound, C_19_H_15_N_5_, the dihedral angles formed by the plane of the triazole ring with those of the 2-pyridyl, 4-pyridyl and *p*-tolyl rings are 28.12 (10), 34.62 (10) and 71.43 (9)°, respectively. The crystal structure is consolidated by C—H⋯π hydrogen-bonding inter­actions and by π–π stacking inter­actions, with a centroid–centroid distance of 3.794 (4) Å.

## Related literature

For the pharmaceutical and agricultural applications of triazoles, see: Grénman *et al.* (2003 ▶). For general background on the coordination chemistry of triazoles, see: Haasnoot (2000 ▶); Klingele & Brooker (2003 ▶); Beckmann & Brooker (2003 ▶). For the synthesis of the title compound, see: Erwin (1958 ▶).
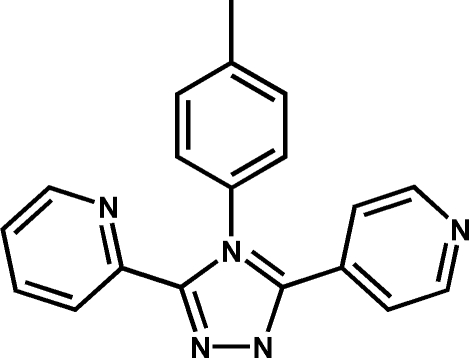


## Experimental

### 

#### Crystal data

C_19_H_15_N_5_
*M* *_r_* = 313.36
Monoclinic, 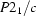

*a* = 5.6104 (11) Å
*b* = 16.312 (3) Å
*c* = 16.902 (4) Å
β = 105.07 (3)°
*V* = 1493.6 (6) Å^3^
*Z* = 4
Mo *K*α radiation
μ = 0.09 mm^−1^
*T* = 293 K
0.20 × 0.20 × 0.20 mm

#### Data collection

Rigaku SCXmini diffractometer
Absorption correction: multi-scan (*CrystalClear*; Rigaku, 2005 ▶) *T*_min_ = 0.982, *T*_max_ = 0.983
13896 measured reflections
2918 independent reflections
1734 reflections with *I* > 2σ(*I*)
*R*_int_ = 0.105

#### Refinement

*R*[*F* ^2^ > 2σ(*F* ^2^)] = 0.071
*wR*(*F* ^2^) = 0.159
*S* = 1.07
2918 reflections
217 parameters
H-atom parameters constrained
Δρ_max_ = 0.32 e Å^−3^
Δρ_min_ = −0.26 e Å^−3^

### 

Data collection: *CrystalClear* (Rigaku, 2005 ▶); cell refinement: *CrystalClear*; data reduction: *CrystalClear*; program(s) used to solve structure: *SHELXS97* (Sheldrick, 2008 ▶); program(s) used to refine structure: *SHELXL97* (Sheldrick, 2008 ▶); molecular graphics: *SHELXTL/PC* (Sheldrick, 2008 ▶); software used to prepare material for publication: *PRPKAPPA* (Ferguson, 1999 ▶).

## Supplementary Material

Crystal structure: contains datablocks I, global. DOI: 10.1107/S1600536809016432/rz2317sup1.cif

Structure factors: contains datablocks I. DOI: 10.1107/S1600536809016432/rz2317Isup2.hkl

Additional supplementary materials:  crystallographic information; 3D view; checkCIF report

## Figures and Tables

**Table 1:** Hydrogen-bond geometry (Å, °)

*D*—H⋯*A*	*D*—H	H⋯*A*	*D*⋯*A*	*D*—H⋯*A*
C11—H11*A*⋯*Cg*1^i^	0.93	2.79	3.630 (4)	150
C12—H12*A*⋯*Cg*3	0.93	2.90	3.532 (4)	126
C14—H14*A*⋯*Cg*1^ii^	0.93	2.76	3.628 (4)	156
C18—H18*A*⋯*Cg*2^iii^	0.93	2.78	3.615 (4)	149
C19—H19*C*⋯*Cg*3^iv^	0.96	3.08	3.698 (4)	124

Symmetry codes: (i) 
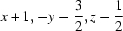
; (ii) 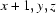; (iii) 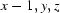; (iv) 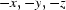. *Cg*1, *Cg*2 and *Cg*3 are the centroids of the N1–N3/C1,C2, N4/C3–C7 and N5/C8–C12 rings, respectively.

**Table 2:** π–π Stacking interaction geometry

Group 1	Group 2	α (°)	DCC (Å)	τ (°)
*Cg*2	*Cg*2^i^	0.0	3.794 (3)	31.30

Symmetry code: (i) 3 − *x*, 1 − *y*, 2 − *z*. α is the dihedral angle between the planes, DCC is the length of the centroid–centroid vector, τ is the angle subtended by the plane normal to CC and *Cg*2 is the centroid of ring N5/C8–C12.

**Fig. 1.**
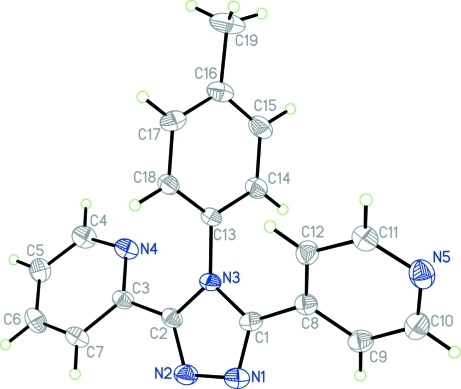
The molecular structure of the title compound, with displacement ellipsoids drawn at the 30% probability level.

## supplementary crystallographic information

### Comment

The main interest in triazoles lies in their pharmaceutical and agricultural applications (Grénman *et al.*, 2003). The utilization of 1,2,4-triazole derivatives as bridging ligands in transition-metal complexes is currently of considerable interest because of the fact that it represents a hybrid of pyrazole and imidazole with regard to the arrangement of its heteroatoms, thus promising a rich and versatile coordination chemistry (Haasnoot, 2000; Klingele & Brooker, 2003; Beckmann & Brooker, 2003). We report here the crystal structure of the title compound, which is a substituted 1,2,4-triazole synthesized by the reaction of 4,4'-dimethylphenylphosphazoanilide with N-(2-pyridyl)-N'-(4-pyridyl)hydrazine in *o*-dichlorobenzene (Erwin, 1958).

The structure of the title compound (Fig. 1) features a dihedral angle of 28.12 (10)° between the 2-pyridyl and triazole rings, a dihedral angle of 34.62 (10)° between the 4-pyridyl and triazole rings, and a dihedral angle of 71.43 (9) ° between the *p*-tolyl and the triazole rings. The crystal structure is stabilized by C—H···π hydrogen interactions (Table 1) and π–π stacking interactions (Table 2).

### Experimental

A mixture of 4,4'-dimethylphenylphosphazoanilide (3.60 g, 14.9 mmol) and N-(2-pyridyl)-N'-(4-pyridyl)hydrazine (3.00 g, 12.4 mmol) in *o*-dichlorobenzene (30 ml) was refluxed for 3 h, then conc. HCl (5 ml) and H_2_O (5 ml) were added to the system after the removal of the solvent under reduced pressure. After refluxing for 1 h, the mixture was filtered and the fietrate was neutralized with K_2_CO_3_ to pH 8–9 to achieve a white solid. Colourless crystals of the title compound suitable for X-ray diffraction were obtained by slow evaporation of an ethanol solution.

### Refinement

Positional parameters of all the H atoms were calculated geometrically and were allowed to ride on the C atoms to which they are bonded with, C—H = 0.93 Å (aromatic) or 0.96 Å (methyl), and with *U*_iso_(H) = 1.2*U*_eq_(C_aromatic_) or *U*_iso_(H) = 1.5*U*_eq_(C_methyl_).

### Crystal data

**Table:**

C_19_H_15_N_5_	*F*(000) = 656
*M**_r_* = 313.36	*D*_x_ = 1.394 Mg m^−^^3^
Monoclinic, *P*2_1_/*c*	Mo *K*α radiation, λ = 0.71073 Å
Hall symbol: -P 2ybc	Cell parameters from 1730 reflections
*a* = 5.6104 (11) Å	θ = 3.0–27.5°
*b* = 16.312 (3) Å	µ = 0.09 mm^−^^1^
*c* = 16.902 (4) Å	*T* = 293 K
β = 105.07 (3)°	Prism, colourless
*V* = 1493.6 (6) Å^3^	0.20 × 0.20 × 0.20 mm
*Z* = 4	

### Data collection

**Table:**

Rigaku SCXmini diffractometer	2918 independent reflections
Radiation source: fine-focus sealed tube	1734 reflections with *I* > 2σ(*I*)
graphite	*R*_int_ = 0.105
CCD_Profile_fitting scans	θ_max_ = 26.0°, θ_min_ = 3.5°
Absorption correction: multi-scan (*CrystalClear*; Rigaku, 2005)	*h* = −6→6
*T*_min_ = 0.982, *T*_max_ = 0.983	*k* = −20→20
13896 measured reflections	*l* = −20→20

### Refinement

**Table:**

Refinement on *F*^2^	Primary atom site location: structure-invariant direct methods
Least-squares matrix: full	Secondary atom site location: difference Fourier map
*R*[*F*^2^ > 2σ(*F*^2^)] = 0.071	Hydrogen site location: inferred from neighbouring sites
*wR*(*F*^2^) = 0.159	H-atom parameters constrained
*S* = 1.07	*w* = 1/[σ^2^(*F*_o_^2^) + (0.0527*P*)^2^ + 0.6603*P*] where *P* = (*F*_o_^2^ + 2*F*_c_^2^)/3
2918 reflections	(Δ/σ)_max_ < 0.001
217 parameters	Δρ_max_ = 0.32 e Å^−^^3^
0 restraints	Δρ_min_ = −0.26 e Å^−^^3^

### Special details

**Table:**

Geometry. All e.s.d.'s (except the e.s.d. in the dihedral angle between two l.s. planes) are estimated using the full covariance matrix. The cell e.s.d.'s are taken into account individually in the estimation of e.s.d.'s in distances, angles and torsion angles; correlations between e.s.d.'s in cell parameters are only used when they are defined by crystal symmetry. An approximate (isotropic) treatment of cell e.s.d.'s is used for estimating e.s.d.'s involving l.s. planes.
Refinement. Refinement of *F*^2^ against ALL reflections. The weighted *R*-factor *wR* and goodness of fit *S* are based on *F*^2^, conventional *R*-factors *R* are based on *F*, with *F* set to zero for negative *F*^2^. The threshold expression of *F*^2^ > σ(*F*^2^) is used only for calculating *R*-factors(gt) *etc*. and is not relevant to the choice of reflections for refinement. *R*-factors based on *F*^2^ are statistically about twice as large as those based on *F*, and *R*- factors based on ALL data will be even larger.

### Fractional atomic coordinates and isotropic or equivalent isotropic displacement parameters (Å^2^)

**Table:**

	*x*	*y*	*z*	*U*_iso_*/*U*_eq_	
C1	1.0349 (6)	0.66367 (18)	0.84606 (18)	0.0362 (8)	
C2	0.7141 (5)	0.73132 (19)	0.78095 (17)	0.0349 (7)	
C3	0.5160 (6)	0.79095 (19)	0.75540 (17)	0.0360 (8)	
C4	0.3738 (7)	0.9211 (2)	0.7518 (2)	0.0508 (9)	
H4B	0.4004	0.9757	0.7673	0.061*	
C5	0.1503 (6)	0.9004 (2)	0.7039 (2)	0.0480 (9)	
H5B	0.0273	0.9396	0.6870	0.058*	
C6	0.1094 (6)	0.8215 (2)	0.6810 (2)	0.0522 (10)	
H6A	−0.0430	0.8056	0.6478	0.063*	
C7	0.2918 (6)	0.7655 (2)	0.70656 (19)	0.0453 (8)	
H7A	0.2658	0.7108	0.6914	0.054*	
C8	1.2577 (6)	0.63716 (17)	0.90639 (19)	0.0346 (7)	
C9	1.4365 (6)	0.59609 (18)	0.8799 (2)	0.0418 (8)	
H9A	1.4153	0.5859	0.8243	0.050*	
C10	1.6455 (6)	0.5704 (2)	0.9356 (2)	0.0471 (9)	
H10A	1.7648	0.5428	0.9167	0.056*	
C11	1.5079 (6)	0.6215 (2)	1.0397 (2)	0.0455 (9)	
H11A	1.5303	0.6301	1.0956	0.055*	
C12	1.2956 (6)	0.64888 (19)	0.98817 (19)	0.0406 (8)	
H12A	1.1774	0.6754	1.0086	0.049*	
C13	0.9579 (5)	0.79279 (17)	0.91387 (17)	0.0310 (7)	
C14	1.1613 (6)	0.84102 (19)	0.92208 (19)	0.0407 (8)	
H14A	1.2539	0.8385	0.8838	0.049*	
C15	1.2267 (6)	0.8930 (2)	0.9874 (2)	0.0481 (9)	
H15A	1.3659	0.9258	0.9935	0.058*	
C16	1.0920 (6)	0.89820 (18)	1.04469 (19)	0.0421 (8)	
C17	0.8873 (6)	0.84945 (19)	1.03423 (19)	0.0429 (8)	
H17A	0.7930	0.8520	1.0720	0.052*	
C18	0.8200 (5)	0.79711 (19)	0.96907 (17)	0.0362 (7)	
H18A	0.6802	0.7645	0.9625	0.043*	
C19	1.1735 (8)	0.9536 (2)	1.1171 (2)	0.0689 (12)	
H19A	1.3198	0.9824	1.1138	0.103*	
H19B	1.2082	0.9216	1.1665	0.103*	
H19C	1.0450	0.9923	1.1176	0.103*	
N1	0.9349 (5)	0.62309 (16)	0.77955 (16)	0.0446 (7)	
N2	0.7292 (5)	0.66575 (17)	0.73835 (16)	0.0443 (7)	
N3	0.9026 (4)	0.73278 (14)	0.84991 (14)	0.0324 (6)	
N4	0.5604 (5)	0.86832 (16)	0.77874 (16)	0.0453 (7)	
N5	1.6862 (5)	0.58304 (17)	1.01564 (19)	0.0508 (8)	

### Atomic displacement parameters (Å^2^)

**Table:**

	*U*^11^	*U*^22^	*U*^33^	*U*^12^	*U*^13^	*U*^23^
C1	0.044 (2)	0.0345 (17)	0.0313 (17)	−0.0008 (15)	0.0120 (15)	−0.0043 (14)
C2	0.0358 (18)	0.0410 (18)	0.0263 (16)	−0.0069 (14)	0.0054 (14)	−0.0005 (15)
C3	0.0404 (18)	0.0421 (19)	0.0238 (15)	−0.0069 (15)	0.0054 (14)	−0.0014 (14)
C4	0.056 (2)	0.040 (2)	0.051 (2)	0.0005 (17)	0.0052 (19)	0.0049 (17)
C5	0.043 (2)	0.050 (2)	0.047 (2)	0.0048 (17)	0.0052 (17)	0.0093 (17)
C6	0.040 (2)	0.062 (3)	0.047 (2)	−0.0066 (18)	−0.0017 (17)	0.0049 (19)
C7	0.046 (2)	0.0430 (19)	0.0419 (19)	−0.0092 (16)	0.0016 (16)	−0.0041 (16)
C8	0.0388 (19)	0.0289 (16)	0.0374 (18)	−0.0048 (13)	0.0122 (15)	0.0015 (14)
C9	0.049 (2)	0.0355 (18)	0.0425 (19)	−0.0032 (16)	0.0153 (17)	−0.0008 (15)
C10	0.048 (2)	0.0399 (19)	0.059 (2)	−0.0016 (16)	0.0227 (19)	0.0037 (17)
C11	0.045 (2)	0.047 (2)	0.042 (2)	−0.0038 (17)	0.0087 (17)	−0.0008 (16)
C12	0.0382 (19)	0.047 (2)	0.0371 (18)	0.0025 (15)	0.0114 (15)	0.0003 (16)
C13	0.0326 (17)	0.0314 (16)	0.0264 (15)	−0.0044 (13)	0.0030 (13)	−0.0009 (13)
C14	0.0410 (19)	0.0423 (19)	0.0382 (18)	−0.0073 (15)	0.0094 (15)	−0.0019 (16)
C15	0.044 (2)	0.0390 (19)	0.055 (2)	−0.0101 (16)	0.0024 (18)	−0.0039 (17)
C16	0.055 (2)	0.0289 (17)	0.0324 (18)	0.0048 (16)	−0.0056 (17)	0.0024 (14)
C17	0.056 (2)	0.0401 (19)	0.0331 (18)	0.0051 (16)	0.0132 (16)	−0.0022 (15)
C18	0.0342 (17)	0.0413 (18)	0.0317 (17)	−0.0066 (14)	0.0060 (14)	−0.0033 (14)
C19	0.098 (3)	0.045 (2)	0.046 (2)	0.005 (2)	−0.013 (2)	−0.0117 (18)
N1	0.0515 (18)	0.0429 (16)	0.0381 (15)	−0.0024 (13)	0.0093 (14)	−0.0070 (13)
N2	0.0477 (17)	0.0464 (16)	0.0360 (15)	−0.0020 (14)	0.0057 (13)	−0.0070 (14)
N3	0.0372 (15)	0.0337 (14)	0.0257 (13)	−0.0043 (11)	0.0070 (11)	−0.0043 (11)
N4	0.0505 (18)	0.0413 (16)	0.0396 (16)	−0.0045 (14)	0.0036 (14)	−0.0006 (13)
N5	0.0429 (17)	0.0485 (18)	0.060 (2)	−0.0045 (14)	0.0115 (15)	0.0045 (15)

### Geometric parameters (Å, °)

**Table:**

C1—N1	1.300 (4)	C10—H10A	0.9300
C1—N3	1.361 (4)	C11—N5	1.331 (4)
C1—C8	1.458 (4)	C11—C12	1.356 (4)
C2—N2	1.304 (4)	C11—H11A	0.9300
C2—N3	1.355 (3)	C12—H12A	0.9300
C2—C3	1.456 (4)	C13—C18	1.360 (4)
C3—N4	1.326 (4)	C13—C14	1.363 (4)
C3—C7	1.377 (4)	C13—N3	1.431 (3)
C4—N4	1.340 (4)	C14—C15	1.365 (4)
C4—C5	1.347 (4)	C14—H14A	0.9300
C4—H4B	0.9300	C15—C16	1.376 (5)
C5—C6	1.345 (5)	C15—H15A	0.9300
C5—H5B	0.9300	C16—C17	1.370 (5)
C6—C7	1.356 (5)	C16—C19	1.494 (4)
C6—H6A	0.9300	C17—C18	1.367 (4)
C7—H7A	0.9300	C17—H17A	0.9300
C8—C12	1.356 (4)	C18—H18A	0.9300
C8—C9	1.375 (4)	C19—H19A	0.9600
C9—C10	1.365 (4)	C19—H19B	0.9600
C9—H9A	0.9300	C19—H19C	0.9600
C10—N5	1.328 (4)	N1—N2	1.372 (4)
			
N1—C1—N3	110.2 (3)	C11—C12—H12A	120.4
N1—C1—C8	123.6 (3)	C8—C12—H12A	120.4
N3—C1—C8	126.3 (3)	C18—C13—C14	120.7 (3)
N2—C2—N3	109.9 (3)	C18—C13—N3	120.2 (3)
N2—C2—C3	122.6 (3)	C14—C13—N3	118.9 (3)
N3—C2—C3	127.5 (3)	C13—C14—C15	118.9 (3)
N4—C3—C7	122.5 (3)	C13—C14—H14A	120.6
N4—C3—C2	118.5 (3)	C15—C14—H14A	120.6
C7—C3—C2	119.0 (3)	C14—C15—C16	121.7 (3)
N4—C4—C5	124.5 (3)	C14—C15—H15A	119.1
N4—C4—H4B	117.8	C16—C15—H15A	119.1
C5—C4—H4B	117.8	C17—C16—C15	117.9 (3)
C6—C5—C4	118.4 (3)	C17—C16—C19	121.6 (3)
C6—C5—H5B	120.8	C15—C16—C19	120.4 (3)
C4—C5—H5B	120.8	C18—C17—C16	120.9 (3)
C5—C6—C7	119.6 (3)	C18—C17—H17A	119.6
C5—C6—H6A	120.2	C16—C17—H17A	119.6
C7—C6—H6A	120.2	C13—C18—C17	119.9 (3)
C6—C7—C3	118.9 (3)	C13—C18—H18A	120.1
C6—C7—H7A	120.5	C17—C18—H18A	120.1
C3—C7—H7A	120.5	C16—C19—H19A	109.5
C12—C8—C9	117.8 (3)	C16—C19—H19B	109.5
C12—C8—C1	123.3 (3)	H19A—C19—H19B	109.5
C9—C8—C1	118.8 (3)	C16—C19—H19C	109.5
C10—C9—C8	119.5 (3)	H19A—C19—H19C	109.5
C10—C9—H9A	120.2	H19B—C19—H19C	109.5
C8—C9—H9A	120.2	C1—N1—N2	107.3 (3)
N5—C10—C9	123.0 (3)	C2—N2—N1	107.6 (2)
N5—C10—H10A	118.5	C2—N3—C1	104.9 (2)
C9—C10—H10A	118.5	C2—N3—C13	129.0 (2)
N5—C11—C12	124.3 (3)	C1—N3—C13	126.1 (2)
N5—C11—H11A	117.9	C3—N4—C4	116.1 (3)
C12—C11—H11A	117.9	C10—N5—C11	116.2 (3)
C11—C12—C8	119.2 (3)		

### Hydrogen-bond geometry (Å, °)

**Table:**

*D*—H···*A*	*D*—H	H···*A*	*D*···*A*	*D*—H···*A*
C11—H11A···Cg1^i^	0.93	2.79	3.630 (4)	150
C12—H12A···Cg3	0.93	2.90	3.532 (4)	126
C14—H14A···Cg1^ii^	0.93	2.76	3.628 (4)	156
C18—H18A···Cg2^iii^	0.93	2.78	3.615 (4)	149
C19—H19C···Cg3^iv^	0.96	3.08	3.698 (4)	124

Symmetry codes: (i) *x*+1, −*y*−3/2, *z*−1/2;  (ii) *x*+1, *y*, *z*; (iii) *x*−1, *y*, *z*; (iv) −*x*, −*y*, −*z*.

### Table 2 π-π Stacking interaction geometry (α is the dihedral angle between the planes, DCC is the length of the centroid–centroid vector, τ is the angle subtended by the plane normal to CC. Cg2 is the centroid of ring N5/C8–C12)

**Table:**

Group 1	Group 2	α (°)	DCC (Å)	τ (°)
Cg2	Cg2^i^	0.0	3.794 (3)	31.30

Symmetry code: (i) 3-x, 1-y, 2-z.
